# Early Neurobehavioral Characterization of the CD Mouse Model of Williams–Beuren Syndrome

**DOI:** 10.3390/cells12030391

**Published:** 2023-01-21

**Authors:** Silvia Giannoccaro, Celeste Ferraguto, Valeria Petroni, Coline Marcelly, Xavier Nogues, Victoria Campuzano, Susanna Pietropaolo

**Affiliations:** 1 Univ. Bordeaux, CNRS, EPHE, INCIA, UMR 5287, F-33000 Bordeaux, France; 2 UF Biologie, Univ. Bordeaux, 33405 Talence, France; 3 Departament de Biomedicina, Universitat de Barcelona, 08007 Barcelona, Spain

**Keywords:** behavioral markers, ultrasonic calls, acoustic startle, neurodevelopmental disorders, CD mice, adolescence, developmental milestones

## Abstract

Williams–Beuren syndrome (WBS) is a neurodevelopmental disorder caused by a chromosomic microdeletion (7q11.23). WBS has been modeled by a mouse line having a complete deletion (CD) of the equivalent mouse locus. This model has been largely used to investigate the etiopathological mechanisms of WBS, although pharmacological therapies have not been identified yet. Surprisingly, CD mice were so far mainly tested in adulthood, despite the developmental nature of WBS and the critical relevance of early timing for potential treatments. Here we provide for the first time a phenotypic characterization of CD mice of both sexes during infancy and adolescence, i.e., between birth and 7 weeks of age. CD pups of both sexes showed reduced body growth, delayed sensory development, and altered patterns of ultrasonic vocalizations and exploratory behaviors. Adolescent CD mice showed reduced locomotion and acoustic startle response, and altered social interaction and communication, the latter being more pronounced in female mice. Juvenile CD mutants of both sexes also displayed reduced brain weight, cortical and hippocampal dendritic length, and spine density. Our findings highlight the critical relevance of early neurobehavioral alterations as biomarkers of WBS pathology, underlying the importance of adolescence for identifying novel therapeutic targets for this neurological disorder.

## 1. Introduction

Williams–Beuren syndrome (WBS) is a rare neurodevelopmental disorder (NDD) equally occurring in both sexes [1,2], caused by the hemizygous deletion of 26–28 genes on chromosome band 7q11.23 [3]. WBS male and female patients present physical abnormalities, including growth retardation [4], a neurological phenotype with motor problems, acoustic alterations, and a characteristic hypersocial profile [5]. A mouse model recapitulating the complete chromosomic deletion (CD mouse) and most of the behavioral phenotypes of WBS has been engineered in 2014 [6]. This preclinical model has largely contributed to advancing our understanding of the brain mechanisms involved in WBS pathology, including reduced brain development, altered synaptic mechanisms, and dendritic abnormalities in selected brain areas [7,8,9,10,11]. The CD model has also been recently employed to test the efficacy of potential novel therapeutic approaches to WBS [12]. This issue is of critical relevance since no pharmacological treatment for the whole neurological phenotype has been identified yet.

All available studies on the CD model have employed exclusively adult mice, i.e., older than 3 months old. The lack of earlier studies represents a serious lacuna for preclinical research on WBS, considering the developmental nature of this syndrome, the early appearance of the physical and neurological phenotypes, and the consequent need for early therapeutic interventions. Furthermore, several research tools are available to investigate early behavioral phenotypes relevant to NDDs in mice [13]: the reaching of developmental milestones (i.e., ear detachment, eye opening, acoustic startle reflex) has been largely employed in mouse models of NDDs [14,15,16], together with the assessment of ultrasonic vocalizations (USVs) that are emitted by pups isolated from their mother and littermates [17,18]. Measuring USVs in particular has emerged as a powerful tool to assess the behavioral validity of mouse models of NDDs characterized by social and communication deficits, e.g., Autism spectrum disorder (ASD) [19]. USVs allow indeed both quantitative and qualitative characterization of early social alterations and can be evaluated not only in infancy but also during adolescence, i.e., in juvenile same-sex mice [19,20,21].

Adolescence is indeed an ontogenetic phase of high relevance for the behavioral characterization of mouse models of NDDs, in particular those including social abnormalities. Social behavior patterns typically emerged during adolescence, ranging in the mouse species between the 3 and 7 weeks of age [22,23]. These patterns are expressed in a highly sex-specific manner, with agonistic behaviors having a peak around 5 weeks of age in males [23] when females mostly display affiliative behaviors [22]. Adolescence is also a life phase of high interest for post-natal brain development, because of the occurrence of dendritic/synaptic pruning and brain remodeling [24]; it also represents the earliest phase allowing easily performing brain analyses as well as therapeutic interventions in laboratory mice, without interfering with mother–pup interactions. For these reasons, a growing number of studies have assessed the behavioral validity of selected mouse models of NDDs, e.g., ASD, in juvenile/adolescent mice (e.g., [25,26,27]). Nonetheless, an adolescent neurobehavioral characterization has never been performed in the CD mouse model of WBS.

Here we provide a characterization of the neurobehavioral phenotype of the CD mouse model of WBS, including both infancy and adolescence. Male and female CD mice and their WT littermates were assessed during the first 3 post-natal weeks, including measures for body growth (weight and length) and developmental milestones (ear detachment, eye opening, and acoustic startle). USVs were also evaluated in mouse pups, together with their exploratory behaviors during testing sessions of isolation from the mother and littermates. All these variables, including physical, behavioral, and vocalization development were combined into a Principal Component Analysis (PCA) to further analyze their potential interactions in mutant and wild-type pups. PCA was applied for each testing day in order to provide additional information about the relationships occurring among the multiple developmental measures. Physical development, exploratory behaviors, and ultrasonic vocalizations are often independently measured in mouse studies, but their concomitant assessment is not a common procedure so their inter-relationships remain largely unknown. Sensori-motor responses were further evaluated in early and late adolescence, i.e., at 3 and 7 weeks of age, in the open field and acoustic startle test. Adolescent mice were also tested for their social behaviors (i.e., affiliation and USVs) towards a conspecific of the same sex and age between 5 and 7 weeks of age, i.e., the most critical adolescent phase for the expression of social patterns in the mouse species. Adolescent mice of this age range were also assessed in a separate brain anatomical study, evaluating brain abnormalities commonly described in adult CD mice [6], i.e., reduced brain weight, reduced dendritic length, and density in cortical and hippocampal areas.

Since our study included mice of both sexes (because of the equivalent occurrence of WBS neurological phenotypes in male and female patients [1,2]), the estrous cycle of female juvenile mice was assessed on each day of behavioral testing. Cycle differences could indeed affect the considered behaviors (exploratory activity, acoustic startle and social interaction, and USVs [28,29]) and they could interfere with genotype differences, in case of a constantly unbalanced distribution of estrous phases between WT and CD mutants.

## 2. Materials and Methods

### 2.1. Animals

Subjects were CD mutant heterozygous mice and their wild-type littermates (maintained on B6 background [6]). A single cohort of a total of 29 males (17 WT and 12 CD mutants) and 27 females (16 WT and 11 CD mutants) were used for the behavioral assessment (Study 1), while 29 males (13 WT and 16 CD mutants) and 28 females (12 WT and 16 CD mutants) were employed for brain analyses (Study 2).

Mice for study 1 were bred in our animal facility at Bordeaux University. Breeding trios were formed by mating two wild-type C57BL/6J females purchased from Janvier (Le Genest St Isle, France) with a CD heterozygous male [6]. Mice used in study 2 were bred and maintained in the animal facility at the University of Barcelona; CD heterozygous mice, obtained as previously described [6] were crossed with Thy1-YFP transgenic mice (B6.Cg-Tg(Thy1-YFPH)2Jrs/J, Jackson Laboratory, Bar Harbor, ME USA) [30] in order to label pyramidal neurons and allow direct evaluation of dendritic length.

On PND 3 pups were marked by paw tattoo, using a non-toxic odor-less ink (Ketchum permanent Tattoo Inks green paste, Ketchum MFG. Co., New York, NY, USA), and tail samples were collected for DNA extraction and subsequent PCR genotype assessment as previously described [6]. Mice were weaned on PND 21 and housed in same-sex cages in groups of 3–5 animals/cage in polycarbonate standard cages (33 × 15 × 14 cm in size; Tecniplast, Limonest, France) with nestlets as cage enrichment and kept in an air-conditioned room (temperature 21 ± 1 °C; humidity 55%) with lights on from 07:00 a.m. to 07:00 p.m.

### 2.2. Study 1: Early Behavioral Characterization of the CD Mouse Model

**Figure 1:**
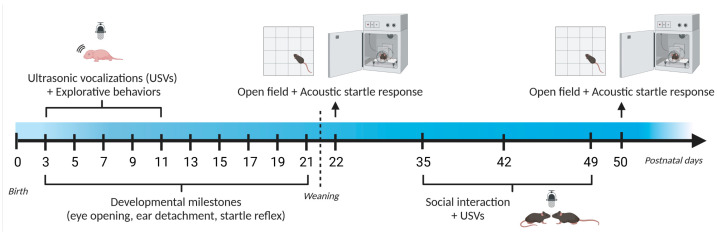
Timeline of behavioral assessment (Study 1). A single batch of CD mice (12 males and 11 females) and their WT littermates (17 males and 16 females) were assessed at infancy (during the first 3 post-natal weeks). The same mouse cohort was tested during adolescence (between 3 and 7 weeks of age). USVs and explorative behaviors were evaluated during a 3-min isolation test of the pups on post-natal days (PNDs) 3, 5, 7, 9, and 11, while body weight/length and developmental milestones were assessed on PND 3, 5, 7, 9, 11, 13, 15, 17, 19 and 21. Mice were weaned at the end of PND 21. Sensori-motor abilities were tested in juvenile mice on PND 22 and 50, while social behaviors were assessed on PND 35, 42, and 49 during 6-min social encounters with a novel WT mouse of the same sex and age.

A single batch of mice was behaviorally assessed first during infancy (starting at PND 3) and then during adolescence (PND 22–50). The precise timeline is illustrated in Figure 1. All behavioral tests were carried out during the light phase of the cycle (between 9 a.m. and 4 p.m.) by an experimenter who was blind to the genotype of the subjects. All mice were habituated to the experimental room for at least 30 min before the beginning of each behavioral test.

#### 2.2.1. Behavioral Assessment of Mouse Pups

USVs were assessed in CD pups and their WT littermates on PND 3, 5, 7, 9, and 11, during a 3-min daily testing session. Pups were individually taken from their nest in a random sequence and placed into a glass container (10 × 8 × 7 cm; open surface), containing clean bedding material (3 cm; room temperature: 22–24 °C). USVs were captured by an UltraSoundGate Condenser Microphone CM 16 (Avisoft Bioacoustics, Glienicke/Nordbahn, Germany) placed 20 cm above the bedding. The microphone was connected via an UltraSoundGate 116 USB audio device (Avisoft Bioacoustics) to a personal computer, where acoustic data were recorded with a sampling rate of 250 kHz in 16-bit format by Avisoft RECORDER (version 4.3 Avisoft Bioacoustics, Glienicke/Nordbahn, Germany). At the end of each testing session, both body weight and length were measured, together with the following developmental milestones: ear detachment, eye opening and the whole-body startle response to a low-intensity click stimulus. Pups were then returned to their nest and left undisturbed until the following testing session (see also Figure 1).

USV recordings were transferred to Avisoft SASLab Pro (Version 5.3; Avisoft) and a Fast Fourier transformation was applied (512 FFT length, 100% frame, Hamming window, and 75% time window overlap). Call detection was provided by an automatic threshold-based algorithm and the accuracy of call detection by the software was verified manually by an experienced user. In addition to the number and mean duration of the USVs, the mean peak amplitude and peak frequency were also computed (i.e., the highest energy within the spectrum of the element and the frequency at the location of the peak amplitude [31]).

During USV recordings, pups’ behavior was recorded by a video camera mounted next to the ultrasonic microphone. Video files were analyzed using observer XT (Version 15, Noldus, Wageningen, The Netherlands) by an experimenter who was blind to the sex and genotype of the subjects. The following behavioral patterns were assessed [32]: head rising (lifting of the head), circling (moving in a circle around itself), wall climbing (alternating movement of forelimbs on the wall of the apparatus), locomotion (body displacement of at least 1 cm in the glass container), probing (pushing the snout against the wall of the container), paddling (movement of forelimbs on the floor of the apparatus without any displacement of the body), roll and curl (side to side rolling movements while on the back and a convex arching of back while on the side), upside down (overturning with the back on the floor of the container), and lying still (no visible movement of the animal). Among these, five behavioral items, i.e., head rising, locomotion, probing, paddling, and lying still, were selected and subjected to statistical analyses, since they represented the major explorative behaviors displayed by pups on all testing days [32]. For each of these behaviors, the frequency was computed and analyzed.

#### 2.2.2. Behavioral Assessment of Juvenile Mice

Juvenile mice were assessed for their sensori-motor abilities in the open field and acoustic startle tests twice, i.e., on PND 22 and 50. Social interaction and communication were evaluated on PND 35, 42, and 49 (see also Figure 1).

- Sensori-motor assessment

Mice were assessed first in a 5-min session of the open field test, consisting of four white opaque plastic arenas (42 × 26 × 15 cm). The total distance traveled was computed through the automated tracking of the videos obtained from a camera above the open field using Ethovision (version 13, Noldus, Wageningen, The Netherlands). One hour after the open field, mice were assessed in the acoustic startle test, as described in detail elsewhere [26]. Briefly, the apparatus consisted of four startle chambers (SR-LAB, San Diego Instruments, San Diego, CA, USA), each allowing the quantification of the whole-body startle response to pulses of white sound of 20 ms duration and varying intensity: +6, +12 +18 and +24 dB over the 66 dB background level (namely 72, 78, 84 and 90 dB). Each intensity was presented 8 times, in a randomized order with variable intervals (10 s to 20 s) between the onset of each pulse.

- Social interaction and USVs

Direct social interaction was assessed in a 33 × 15 × 14 cm plastic cage with 3 cm of sawdust and a metal flat cover. Male experimental subjects were habituated to the apparatus for 15 min prior to testing, while female subjects were isolated in the testing cage for 72 h; the latter procedure was necessary in order to induce a status of resident in female experimental subjects and therefore promote the emission of USVs towards an adult female intruder [33,34]. An unfamiliar stimulus WT mouse of the same sex and age was then introduced into the testing cage of either male or female juvenile subjects and left there for 6 min. In line with previous studies in mouse models of NDDs, stimulus mice were wild-type C57BL/6J male and female juvenile mice (5 weeks of age at the first testing session), purchased from Janvier (Le Genest St Isle, France) and housed in groups of 4–5 animals/cage under the same conditions described before for experimental animals. Each experimental subject encountered a novel stimulus mouse at each testing session, i.e., on PND 35, 42 and 49.

Testing sessions were recorded by a camera placed on the side of the cage and videos were analyzed with Observer XT (version 15, Noldus, Wageningen, The Netherlands). One observer who was unaware of the genotype and sex of the animals scored the behavior only of the experimental subjects, quantifying the time spent performing affiliative behaviors, i.e., sniffing and allogrooming the stimulus mouse. Nonsocial activities, including self-grooming, wall-rearing, and digging, were also scored as described in detail before [26,35].

During each session of social interaction, USVs were recorded by the ultrasonic microphone previously described, which was mounted 2 cm above the cover of the testing cage. Recordings were then transferred to the Sonotrack Call Classification Software (version 1.4.7, Metris B.V., Hoofddorp, The Netherlands). This software fully automatically recognizes different USV types and calculates quantitative parameters including the total number and mean duration of the calls. Based on the previous literature on call types [19,36], the following USV types were selected for automatic recognition in our dataset: Short, Flat, (Ramp) Up, (Ramp) Down, Chevron, Step-Up, Step-Down, Step-Double (Split), Complex-3, Complex-4, Complex-5, Complex-5+. Their characteristics are described in detail in [31,37,38].

- Assessment of female estrous cycle

The estrous phase of juvenile CD and WT females was assessed by the analysis of vaginal smears [39] performed at the end of each testing day.

### 2.3. Study 2: Early Brain Characterization of the CD Mouse Model

A separate batch of 57 mice (13 WT and 16 CD males, 12 WT and 16 CD females) was employed for this study. These mice were behaviorally naive and were kept undisturbed until the age of 5–7 weeks. After sacrifice animals were perfused with 1X PBS followed by 4% PFA. Brains were removed and weighed. After postfixed steps (at 4 °C, 24 h 4% PFA; 24 h 1X PBS), brains were cryoprotected in a 30% sucrose solution for at least 24 h before being cut. Then, 150 μm thick serial coronal brain sections were collected on a glass slide and directly mounted with Mowiol. For dendrite length analysis, 1360 × 1024 pixel images of CA1 hippocampus and motor cortex were obtained with an Olympus DP71 camera attached to an Olympus BX51 microscope with an Olympus U-RFL-T source of fluorescence at 10× magnification. For spine density analyses, 1024 × 1024 pixel confocal fluorescent image stacks from these tissue sections were obtained with a TCS SP2 LEICA confocal microscope, using an X63 (zoom ×5) oil immersion objective. We obtained pictures of dendritic segments of 15–30 µm from randomly selected neurons in CA1 hippocampus sections. Spine counts included all types of dendritic protrusions. Spine density was calculated by relativizing the total number of spines to the length of the analyzed dendrite. Image J software was used for quantification. Ten dendritic segments were analyzed per animal.

### 2.4. Statistical Analysis

Data were analyzed by an ANOVA with genotype and sex as the between-subject factors and testing days as the within-subject factor (repeated measures). Post-hoc pairwise comparisons were performed when a significant interaction was detected. Pulse intensities were included in the analysis of the acoustic startle data as an additional within-subject factor. Only the data from the juvenile social interaction tests were analyzed separately in the two sexes through a genotype x testing day ANOVA, because of the different experimental settings required for testing mice of each sex, i.e., different duration of pre-testing isolation. For developmental milestones (eye opening, ear detachment and startle reflex), contingency tables (crossing genotype and first day of behavior) were analyzed through Fisher exact test (Table 1). The latter statistical approach was applied also to evaluate potential differences in the estrous cycle distribution in adolescent females (see Table 2) and the presence of aggressive episodes during social interaction in adolescent males. Principal component analysis (PCA) was performed to the pups’ variables (body weight and length, USV parameters and frequency of explorative behaviors) on each PND with R software, packages “FactoMineR” and “factoextra”.

All other analyses were conducted using the software Statview (SAS institute, 5.0.1, Cary, NC, USA) and SPSS (PAWS Statistics 18, Chicago, IL, USA) and α was set at 0.05. Results are expressed as mean ± SEM throughout the text. The exact number of mice is indicated in the legend of each figure; slight differences across tests may be due to technical reasons (e.g., loss of video or audio recordings; interruption of the testing session for aggressive episodes or non-vocalizing mice).

## 3. Results

### 3.1. Study 1: Early Behavioral Characterization of the CD Mouse Model

#### 3.1.1. Behavioral Assessment of Mouse Pups

- Body weight/length and developmental milestones

**Figure 2:**
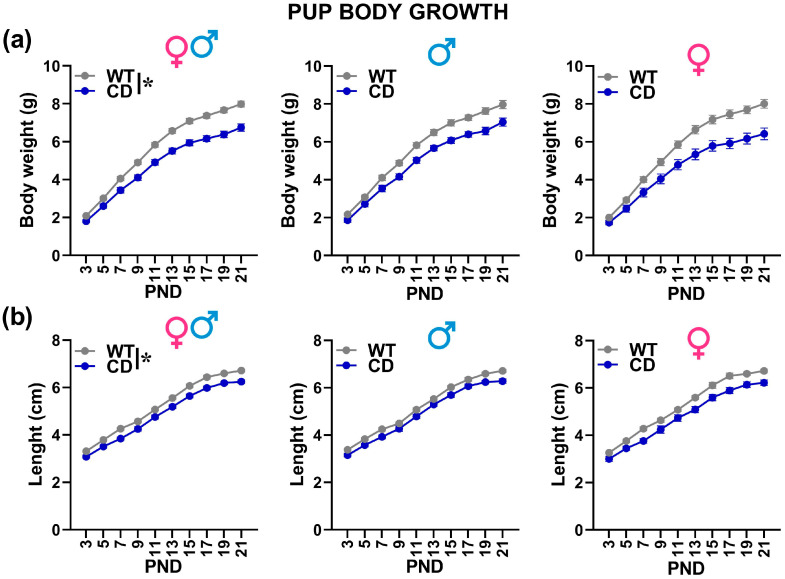
Body growth of mouse pups. Body weight (**a**) and length (**b**) were assessed during the first 3 post-natal weeks. * = *p* < 0.05 referring to a main genotype effect. Statistical analyses were not conducted separately in males and females since no significant interaction with sex emerged. *n* = 29 males (17 WT, 12 CD) and 27 females (16 WT, 11 CD).

Irrespectively of sex, CD mutant mice were characterized by lower body weight and length [genotype effect: F(1,52) = 26.85 and 27.38; *p* < 0.0001; Figure 2a,b], an effect that was already significant on PND 3 and became more pronounced with time [Genotype × testing day: F(9,468) = 19.48 and 3.17; *p* < 0.0001 and *p* = 0.001; Figure 2].

Concerning the developmental milestones (i.e., first appearance of ear detachment, eye opening and acoustic startle response), no significant difference was found between WT and CD groups (*p* > 0.1) for ear detachment and eye opening (Table 1), with 100% of mice reaching these milestones, respectively, on PND 7 and 21, respectively. Acoustic startle response appeared instead later in CD mice irrespectively of their sex, i.e., a larger proportion of WT showing acoustic startle reflex on PND 15 compared to approximately half of the CD mutants (Fisher test on PND 15, CD versus WT: *p* < 0.05, Table 1).

- USVs

**Figure 3:**
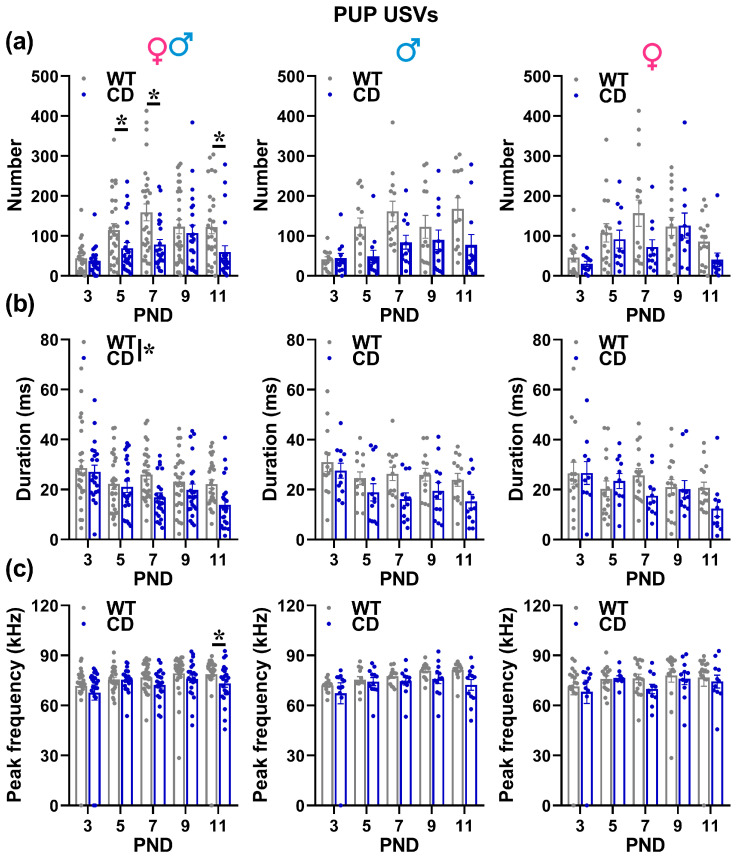
Ultrasonic vocalizations (USVs) emitted by mouse pups. USVs were recorded by isolated pups during 3-min sessions and analyzed in their number (**a**), mean duration (**b**) and mean peak frequency (**c**) using Avisoft software, as described in the text. * = *p* < 0.05. Genotype differences emerged from ANOVA either from a main significant genotype effect (**b**) or from a significant interaction genotype × testing day followed by pairwise comparisons (**a**,**c**). Statistical analyses were not conducted separately in males and females since no significant interaction with sex emerged. *N* = 25 males (13 WT, 12 CD) and 27 females (16 WT, 11 CD).

Irrespectively of pups’ sex, the number of USVs followed a clear time-dependent pattern, increasing from PND 3 to reach a peak around PND 7 to decrease afterwards [testing day effect: F(4,192) = 9.47, *p* < 0.0001; Figure 3a]. This developmental profile was altered in CD mutants irrespectively of their sex, showing a delayed peak in the number of USVs and a more marked decrease with time, emitting fewer USVs than their WT littermates on PND 5, 7 and PND 11 [genotype × testing day: F(4,192) = 2.51, *p* = 0.043; Figure 3a]. The mean USV duration also changed with time, i.e., it decreased progressively from PND3 to PND 11 [testing day effect: F(4,176) = 6.29, *p* < 0.0001; Figure 3b]; this effect tended to be more pronounced in CD mutants, emitting overall shorter USVs than their WT littermates [Genotype effect: F(1,44) = 7.36, *p* = 0.0095; Figure 3b].

CD mutants also showed lower values of peak frequency, but this appeared later, i.e., on PND 11 [genotype × testing day: F(4,176) = 2.87, *p* = 0.0245; Figure 3c]. No difference between sexes or genotypes was found on peak amplitude (data not shown).

- Explorative behaviors

**Figure 4:**
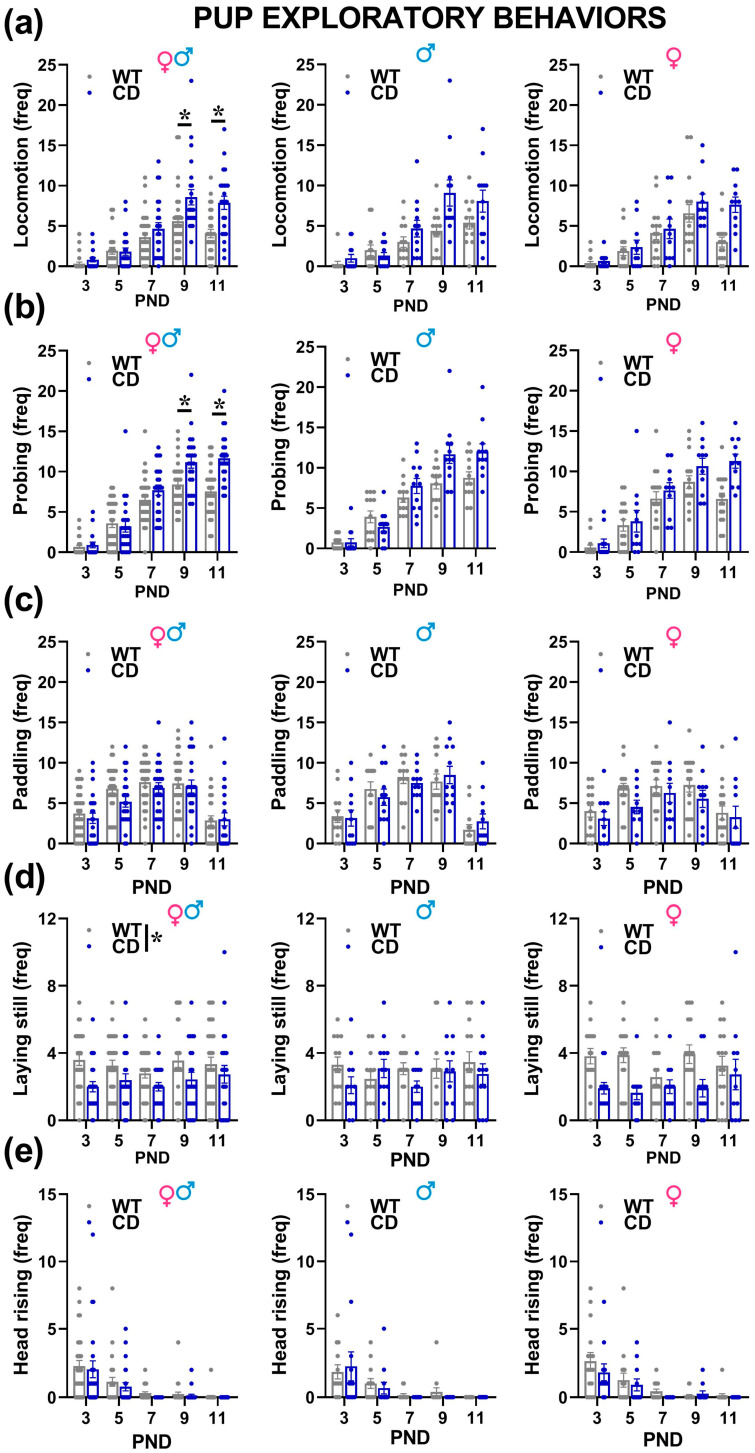
Exploratory behaviors displayed by mouse pups. During USV recording pups’ behaviors were assessed by the analysis of video files and their frequency was scored. The following behaviors were mostly displayed on all PNDs: locomotion (**a**); body displacement of at least 1 cm in the glass container, probing (**b**); pushing the snout against the wall of the container, paddling (**c**); movement of forelimbs on the floor of the apparatus without any displacement of the body, lying still (**d**); no visible movement of the animal and head rising I; lifting of the head, and * = *p* < 0.05. Genotype differences emerged from ANOVA either from a main significant genotype effect (**e**) or from a significant interaction genotype × testing day followed by pairwise comparisons (**a**,**b**). Statistical analyses were not conducted separately in males and females since no significant interaction with sex emerged. *n* = 25 males (13 WT, 12 CD) and 27 females (16 WT, 11 CD).

Irrespectively of their sex, CD mutant mice showed alterations almost in all the explorative behaviors analyzed during USV recordings (Figure 4). Locomotion and probing increased with time [testing day effect: F(4,192) = 42.76 and 100.51, *p* < 0.0001, Figure 4a,b] with a peak around PND 9; this effect tended to be more marked in CD male and female mutants displaying more often locomotion and probing on PNDs 9 and 11 [genotype × testing day: F(4,192) = 3.94 and 5.18, *p* = 0.0042 and *p* = 0.0006; Figure 4a,b].

No genotype difference was instead detected on paddling that tended also to increase with time and to reach a peak on PND 9 [Day effect: F(4,192) = 22.03, *p* < 0.0001, Figure 4c]. The higher expression of explorative behaviors was mirrored by reduced levels of laying still in CD mice [genotype effect: F(1,48) = 14.68, *p* = 0.0004; Figure 4d]. No genotype difference emerged on head rising, a behavior that tended to decrease with time [testing day effect: F(4,192) = 21.16, *p* < 0.0001, Figure 4e].

- PCA analysis

**Figure 5:**
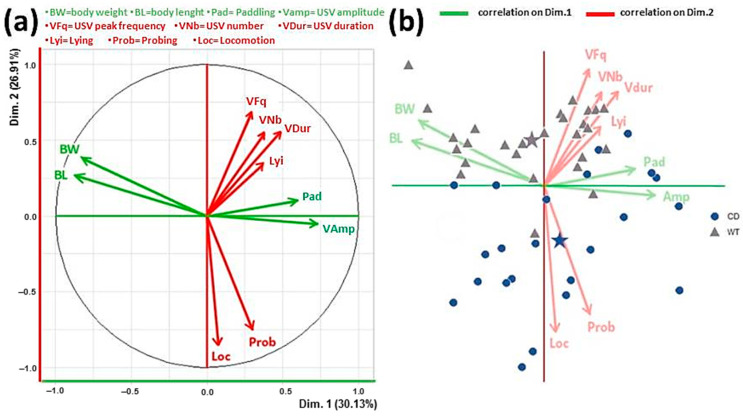
Principal component analysis (PCA) of pup data. Factorial plane (**a**) representing axes 1 (horizontal) and 2 (vertical). Data illustrated here were obtained only on PND 11, i.e., when the higher variability emerged, but they were confirmed by PCA performed on all other testing days. The horizontal axis was mainly correlated with morphological data (weight and length; (**a**)-left) which were negatively correlated with paddling behavior and vocalization peak amplitude ((**a**)-right). The vertical axis was correlated with locomotion and probing behaviors ((**a**)-down) which were negatively correlated with vocalization peak frequency, duration, and number ((**a**)-up). Lying belonged to the latter cluster of variables and was unsurprisingly negatively correlated with locomotion and probing. Representation of data points from all mice on the variable space (**b**). Stars indicate the barycenter of the data clouds from subjects of the two genotypes. Panel b shows the effects of CD mutation, confirming the results of the ANOVAs on the single variables. The discrimination between the two genotypes occurred along the second axis (dimension 2), CD mice being characterized by lower body weight and length, less and shorter USVs with lower peak frequency, displaying less locomotion and probing.

PCA analysis was performed separately on each PND in order to evaluate the potential interactions occurring among the multiple behavioral, physical and vocalization-related variables. The PCA factorial plane representing axes 1 and 2 displays 57% of variance (Figure 5a). The first axis (horizontal axis) was mainly correlated with morphological data (weight and length; Figure 5a left) which were negatively correlated with paddling behavior and vocalization peak amplitude (Figure 5a right). The second axis (vertical) was correlated with locomotion and probing behaviors (Figure 5a down) which were negatively correlated with vocalization frequency, duration and number (Figure 5a up). Lying belonged to that cluster of variables and was unsurprisingly negatively correlated with locomotion and probing. Axes 3 and 4 both capitalized about 10% variance each (eigenvalues about 1) and were not clearly associated with given clusters of variables. This pattern of results was consistently observed on each testing day, although Figure 5 illustrates the most representative dataset, i.e., PND 11, characterized by the highest levels of variability.

These correlations emerged overall in mice of both genotypes. Interestingly, the representation of mice on the variable space, according to their genotype clearly showed the effect of mutation (Figure 5b): CD mice being characterized by lower body weight and length, less and shorter USVs with lower peak frequency, displaying less locomotion and probing. The discrimination between the two groups occurred along axis number 2.

#### 3.1.2. Behavioral Assessment of Juvenile Mice

- Sensori-motor assessment

**Figure 6:**
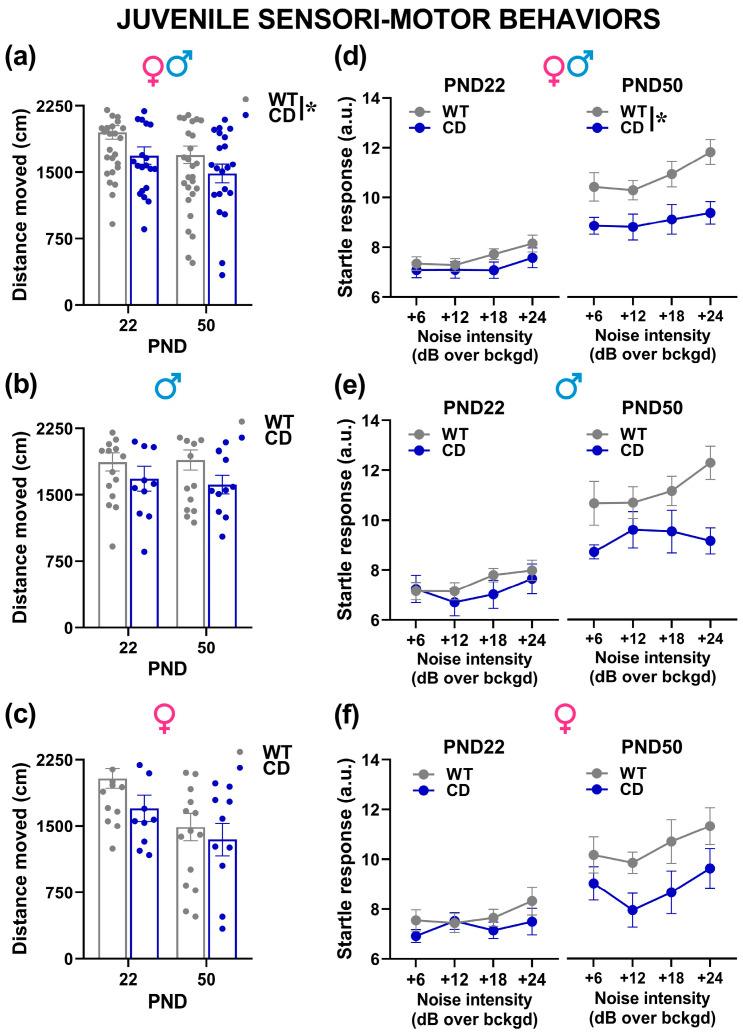
Sensori-motor assessment of juvenile mice. Locomotion was evaluated in the open field (**a**–**c**), followed on the same testing day by an acoustic startle test (**d**–**f**). a.u.= arbitrary units. * = *p* < 0.05. Genotype differences emerged from ANOVA either from a main significant genotype effect (**a**) or from a significant interaction genotype × testing day followed by pairwise comparisons (**d**). Statistical analyses were not conducted separately in males and females since no significant interaction with sex emerged. *n* = 28 males (17 WT, 11 CD) and 26 females (16 WT, 10 CD).

Irrespective of sex, CD mutant mice moved less than their WT littermates in the open field on both testing days [genotype effect: F(1,50) = 6.11, *p* = 0.017; Figure 6a]. Locomotion tended to decrease from PND 22 to PND 50 [testing day effect: F(1,50) = 6.01, *p* = 0.018], although this phenomenon was mostly observed in female mice [sex x testing day: F(1,50) = 4.96, *p* = 0.03; Figure 6c]. No other effect of sex was detected on locomotion in the open field test [sex effect and its interactions with genotype, all n.s.; Figure 6b,c].

As expected, the startle response of all mice increased with the intensity of the acoustic stimulus [noise intensity effect: F(3,150) = 7.52, *p* = 0.0001; Figure 6d] and with age [testing day effect: F(1,50) = 52.99, *p* < 0.0001; Figure 6d]. CD mutant mice, irrespectively of sex, showed a reduced startle response than their WT littermates [genotype effect: F(1,50) = 10.13, *p* = 0.003; Figure 6d], and this effect was more marked on PND 50 [interaction genotype x testing day: F(1,50) = 4.12, *p* < 0.047; post-hoc: *p* < 0.05; Figure 6d]. No difference was found between the two sexes [sex effect and all its interactions, all n.s.; Figure 6e,f].

- Social interaction and USVs

**Figure 7:**
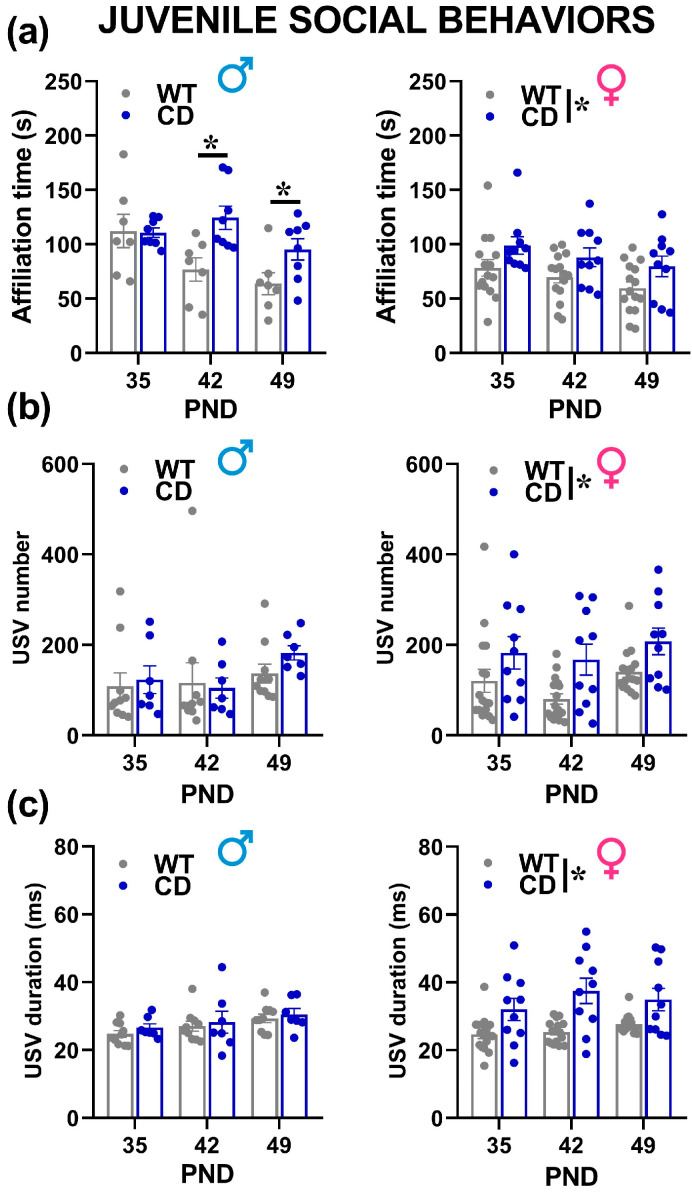
Social interaction and ultrasonic communication in juvenile mice. Affiliative behaviors (**a**), i.e., sniffing and exploring the stimulus mouse, were scored during three 6 min encounters with a conspecific of the same sex and age. Ultrasonic vocalizations (USVs) were analyzed both in their number (**b**) and mean duration (**c**). * = *p* < 0.05. Genotype differences emerged from ANOVA either from a main significant genotype effect ((**a**–**c**) in females) or from a significant interaction genotype x testing day followed by pairwise comparisons ((**a**) in males). Statistical analyses were conducted separately in males and females because different testing procedures were necessary to assess USVs in the two sexes. *n* = 15 males (7 WT, 8 CD) and 26 females (16 WT, 10 CD) for social interaction (**a**); *n* = 17 males (10 WT, 7 CD) and 26 females (16 WT, 10 CD) for USVs (**b**,**c**).

The testing session had to be interrupted for some male animals because of aggression towards the stimulus juvenile male mouse, in order to avoid risks for animals’ welfare. Interestingly, this aggressiveness was mostly observed on PND 35 and 42, and it seemed more frequently displayed by WT males (7 WT males over a total of 16 vs. 3 CD over a total of 10 had to be excluded from the data analysis of social interaction), although this genotype difference failed to reach statistical significance (Fisher exact test, n.s.), sociability tended to decrease from PND 35 to PND 49 in mice of both sexes, as suggested by the decrease in the time spent in affiliative behaviors across testing days [testing day effect in males and females: F(2,26) = 6.33, F(2.46) = 6.41, *p* = 0.006 and *p* = 0.004, respectively; Figure 7a]. This phenomenon was absent in CD mutant male mice, showing enhanced affiliation levels compared to their WT littermates on PND 42 and 49 [genotype x testing day in males: F(2,26) = 3.77, *p* = 0.037; post-hoc:, *p* < 0.05; Figure 7a]. Female CD mutants showed instead overall enhanced affiliation levels on all testing days [genotype effect in females (F(1,23) = 5.12, *p* = 0.033; genotype x testing day: F(2,46) = 0.03, n.s.; Figure 7a]. No difference was found in nonsocial behaviors (data not shown).

**Figure 8:**
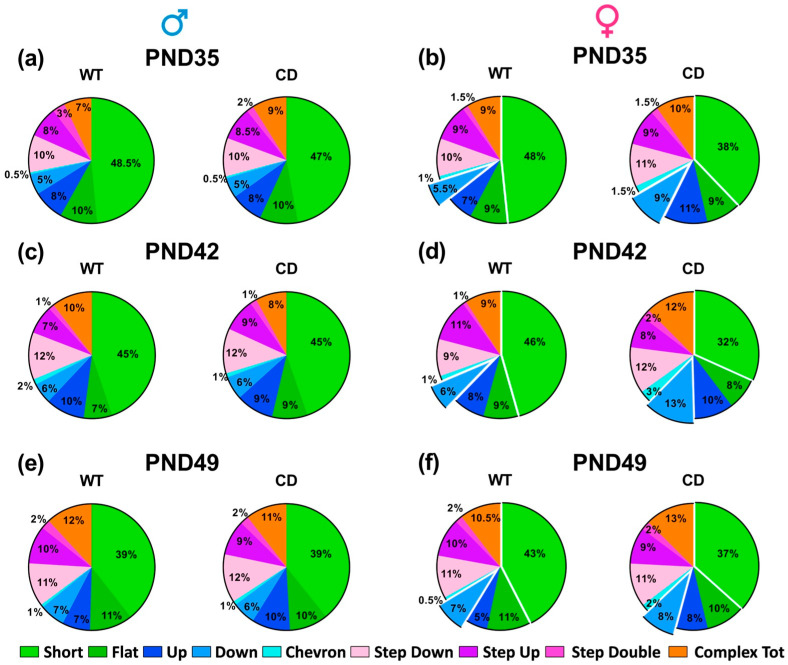
Call type composition of USVs emitted by juvenile mice. Call types were categorized using the software Sonotrack as described in detail elsewhere [31,37,38]. PND = post-natal day. Call categories that differed in CD females (**b**,**d**,**f**) are marked as detached from the remaining types. No differences were detected in males (**a**,**c**,**e**).

Ultrasonic communication was marginally modulated by the testing day: the mean duration of the USVs increased with days, but in males only [testing day effect in males: F(2,30 = 4.77, *p* = 0.0159; in females: F(2,48) = 2.38, ns; Figure 7c]. The other quantitative and qualitative characteristics of USVs were mostly stable across days, including the number and call type composition, where no main effect of the testing day emerged in mice of both sexes (Figure 7b and Figure 8). Independently of the testing day, CD female mice emitted more and longer USVs than their WT littermates [genotype effect in females: F(1,24) = 8.07 and 14.59, *p* = 0.009 and *p* = 0.0008; Figure 7b,c], while no difference emerged in males [genotype effect: F(1,15) = 0.22 and 0.62, n.s.; Figure 7b,c]. The composition of USVs in terms of call types was also altered in CD females: mutants emitted overall less “short” and more “down” calls compared to their WT littermates, at all testing points [genotype effect in females: F(1,24) = 9.30 and 7.83, *p* = 0.005 and *p* = 0.01; Figure 8b,d,f]. No difference emerged in male mice (Figure 8a,c,e).

- Assessment of female estrous cycle

The estrous phase of juvenile CD and WT females was assessed by the analysis of vaginal smears [39] performed at the end of each testing day. The distribution of the estrous phases did not differ between genotypes on any considered day (Fisher exact test on PND 35, 42, 49, and 50, CD versus WT: n.s., Table 2).

### 3.2. Study 2: Early Brain Characterization of the CD Mouse Model

**Figure 9:**
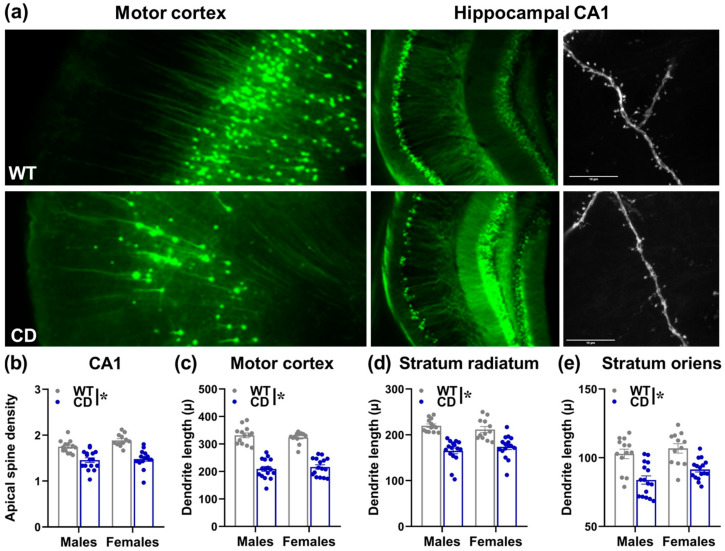
Brain abnormalities in juvenile CD mutant mice. Representative pictures of dendrites in motor cortex and CA1 areas (scale bar 10 µ) of WT and CD mice (**a**). Dendritic spine density (**b**), dendritic length (**c**–**e**), were assessed in CD mice and their WT littermates at the juvenile age (i.e., 5–7 weeks). A separate batch of behaviorally naïve animals was employed for this study. * = *p* < 0.05 refers to the main effect of genotype *n* = 29 males (13 WT, 16 CD) and 28 females (12 WT, 16 CD).

Brain assessment was performed in juvenile male and female mice (i.e., aged between 5 and 7 weeks). Irrespectively of sex, CD mice were characterized by lower brain weights [genotype effect: F(1,53) = 103.77, *p* < 0.0001; Mean ± SEM for WT and CD males (gr) were 0.449 ± 0.004 and 0.388 ± 0.005; for WT and CD females: 0.452 ± 0.004 and 0.401 ± 0.007] and dendritic alterations (Figure 9a). These included reduced dendritic spine density in the CA1 area [genotype effect: F(1,53) = 56.93, *p* < 0.0001; Figure 9b]. Shorter dendritic lengths were also found in CD animals of both sexes in the motor cortex, Stratum Radiatum and Stratum Oriens [main genotype effect: F(1,53)= 192.48, 34.62, 60.91, respectively; *p* < 0001; Figure 9c–e]. No effect of sex or its interaction with genotype was detected on any measured parameter.

## 4. Discussion

Our findings provide for the first time a characterization of CD mice of both sexes during infancy and adolescence, i.e., between birth and 7 weeks of age. CD pups of both sexes showed reduced body growth and displayed altered patterns of ultrasonic vocalizations and explorative behaviors. The appearance of the acoustic startle reflex was also slightly delayed in CD male and female mutants, while other developmental milestones, i.e., eye opening and ear detachment were unaltered. Adolescent CD mice showed reduced locomotion and acoustic startle response, and altered social interaction and communication, the latter being more marked in female mice. Juvenile CD mutants of both sexes also displayed reduced brain weight, and hippocampal and cortical spine length together with reduced spine density in CA1 pyramidal neurons.

The alterations in USVs observed in CD pups of both sexes included first a delayed peak in the number of calls that were observed on PND 9 instead of PND 7 as in WT, followed by a more marked decrease on PND 11 (Figure 3). Second, the USVs emitted by CD pups were overall shorter and with lower peak frequencies on PND 11. These findings are in line with observations from WBS children, often showing a delayed onset of language development [40]. It has been proposed that such linguistic delay may be related to motor abnormalities [41], a hypothesis that may find support in our mouse data. Our PCA analysis (Figure 5) indeed showed an inverse correlation among locomotion and probing, i.e., two major explorative/motor behaviors displayed by mouse pups, and USV number, mean duration, and peak frequency, i.e., all parameters that were altered in CD pups (Figure 4). This finding could open novel interpretations of USV impairments in mouse models of NDDs: a similar delayed USVs expression has been in fact described in genetic mouse models of ASD (e.g., [14,42,43]), but has been rarely associated with concomitant analyses of pup motor behaviors. It is therefore possible that alterations in locomotion or probing may accompany USVs quantitative and qualitative deficits in mutant mice modeling NDDs, and could therefore provide a valuable additional biomarker for their early phenotyping (as previously suggested [32]). Our results also support the utility of PCA analysis in behavioral studies on developing mutant mice, suggesting its potential contribution to revealing novel relationships among multiple behavioral and physical measures.

The delay in USV expression shown by CD mutants was associated with a slight delay in the expression of the acoustic startle reflex that was mostly observed on PND 17 in mutants instead of PND 15 as in WT mice. This sensory developmental delay could be linked to the delayed physical development of CD pups since mutant mice of both sexes were characterized by markedly reduced body weight and length (Figure 2). Notably, the weight deficits were detectable at our earliest considered time-point, i.e., PND 3, and became more pronounced with time. Once again, this finding was in agreement with data from WBS patients describing growth deficits and developmental delays [44].

The acoustic startle was a behavioral phenotype sensitive to CD mutations also at adolescence (Figure 6): CD male and female mutants showed a reduced startle response at late adolescence, i.e., at PND 50, a phenotype that has been described in adult CD mice [45]. This phenotype may resemble the hearing loss often described in WBS patients [46,47,48]; although this acoustic deficit was not evident at PND 22, the low levels of startle response of WT mice at this age could result in a floor effect and therefore mask potential earlier deficits. Additional measures of acoustic responsiveness, e.g., electrophysiological recordings of auditory brainstem potentials, may be useful in the future to confirm our startle finding. These measures have been indeed instrumental in assessing the acoustic phenotypes of CD mice in adulthood [49]. At PND 22 deficits in the open field were already detectable in CD mutants of both sexes, as previously reported in adulthood [45] where they add to motor coordination deficits in the rotarod test [6,45]. Interestingly, the hypoactive profile displayed by CD mice during adolescence suggests a potential phenotypic switch from infancy, when hyperactivity characterized CD pups on PND 9 and 11 (Figure 4a). Both locomotor alterations may reflect motor coordination deficits, although the reasons for the phenotypic switch from infancy to adolescence remain elusive. Altogether these findings support the relevance of adolescence as a critical early phase for the expression of WBS-like sensori-motor deficits in the CD mouse line.

Additional striking WBS-like phenotypes displayed by adolescent CD mice were found in the social domain (Figure 7); here CD mutant males and females displayed higher levels of affiliation that became more evident with testing days. Communication was also altered in CD females (Figure 7 and Figure 8), emitting more and longer USVs, with a higher prevalence of down calls compared to WT female mice. Intriguingly, a similar phenotype, with longer and more abundant USVs, was described in adolescent *Fmr1* mutants, a mouse model of another NDD, and again only in the female sex [26,38]. The female communication phenotype could be due to sex differences in the adolescent patterns of social behaviors: in males, the expression of aggressive behaviors was detected starting on PND 35, as expected in the mouse species [22,23], and could somehow interfere with the expression of USVs. It is indeed interesting to underline that such aggressive tendencies were less often displayed in CD than in WT males, suggesting an altered social development or acquisition of social competence [23]. Nonetheless, additional measures of aggressive behaviors (e.g., latency to attack, frequency of aggressive postures) would be needed to fully investigate the aggressive phenotype of adolescent CD mice, using specific testing protocols for aggression (e.g, resident–intruder tests). The hypersocial phenotype observed here in adolescent CD mutants seemed more marked and robust than the adult one, as the latter was mostly limited to deficits in social novelty preference or social habituation [12,45]. Hence, the adolescent age confirms itself as a highly valuable life phase to assess the WBS-like hypersocial phenotype of the CD model. Altogether, the behavioral phenotypes described in our adolescent mutants reconcile with the existing literature on sensori-motor and social alterations in adult CD mice [12,45].

The relevance of the adolescent phase for the detection of WBS-like phenotypes was not limited to the behavioral domain: marked dendritic abnormalities, more specifically shorter dendritic lengths, and reduced spine density were detected in adolescent CD mice in the cortical and hippocampal areas (Figure 9). Once again, these pathological phenotypes were observed in mutants of both sexes; they emerged in brain areas (motor cortex, dorsal hippocampus) that are involved in the modulation of motor, sensory and social behaviors. These brain abnormalities were previously described in the brains of adult CD mice [8], where they were linked to synaptic plasticity deficits [7], and therefore represent a robust brain marker that may be employed to assess therapeutic interventions together with the WBS-like behavioral phenotypes.

In conclusion, the presence of most neurobehavioral phenotypes in both males and females adds to the translational validity of the CD mouse model, since this NDD is equally observed in patients of both sexes [1,2]. Nonetheless, the ultrasonic communication alterations were found only in female mutants here, underlying the importance of including females in studies on this mouse model. This point is of critical relevance, since several studies on mouse models of NDDs still focus exclusively on male subjects, despite the existing guidelines encouraging the use of animals of both sexes in preclinical research [50]. This prejudice is mostly related to the supposed problem of the estrous cycle in females that is mostly perceived as a potential bias for the results and is often not assessed or controlled. Our results minimize the problematic potential of the estrous cycle issue while encouraging its systematic assessment (in line with previous reports [51]). Future studies may provide additional data on potential correlations between estrous phases and the behavioral phenotype of CD female mice, an issue that would require a larger sample size than the one used in our study. As described in Table 2, genotype differences here could not be ascribed to differences in the estrous cycle, since the estrous cycle was not systemically different between mutant and WT females at any testing point. Furthermore, the scattered attribution of estrous phases allowed excluding that the results obtained here could be idiosyncratic of a specific estrous phase and could therefore complicate the generalization of the results and their replicability. Hence, our neurobehavioral results support and encourage the inclusion of female mice in preclinical studies on WBS and in general on NDDs.

## 5. Conclusions

Our results provide evidence for early neurobehavioral phenotypes in the CD mouse model for WBS, highlighting the relevance of including adolescence and both sexes in future studies on this rare NDD. These findings constitute a solid basis for designing novel therapeutic approaches for this and other neurodevelopmental syndromes, allowing the targeting of early pathological phases.

## Figures and Tables

**Table 1:** Developmental milestones. Number indicates the cumulative percentage of animals of each experimental group reaching the developmental milestones on the corresponding days. * *p* < 0.05: on PND 15, WT versus CD mice (males and mutants pooled together).

GT	Sex	Eye Opening					Ear Detachment					Acoustic Startle		
		PND 15	PND 17	PND 19	PND 21		PND 3	PND 5	PND 7			PND 15	PND 17	PND 19
WT	M	23.5	88.2	94.1	100		35.3	100	100	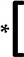	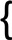	76.5	100	100
	F	37.5	93.8	100	100		25	93.8	100			62.5	100	100
CD	M	33.3	83.3	91.7	100		25	100	100		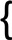	50	100	100
	F	18.2	54.5	81.8	100		9.09	90.9	100			54.5	90.9	100

**Table 2:** Estrous cycle distribution of female mice during juvenile testing. Estrous cycle was assessed after each testing session (for PND 22 and PND 50 after the open field and acoustic startle tests; for PND 35, 42, and 49 after the social interaction tests). On PND 22 no estrous phase was detectable because of the sexual immaturity of all females.

Cycle		PND 22			PND 35			PND 42			PND 49			PND 50	
		WT	CD		WT	CD		WT	CD		WT	CD		WT	CD
PROESTRUS		-	-		6.3	0		0	0		19	0		44	40
ESTRUS		-	-		31	20		13	10		19	40		25	10
METAESTRUS		-	-		13	30		31	0		6.3	0		0	0
DIESTRUS		-	-		50	50		56	90		56	60		31	50

## Data Availability

The datasets used and analyzed during the current study are available from the corresponding author upon reasonable request.
